# Heterogeneity in CT-Based Skeletal Muscle Mass Assessment and Sarcopenia Diagnosis Undermines Comparability in Hepatopancreatobiliary Surgery: A Scoping Review

**DOI:** 10.3390/jcm15207856

**Published:** 2026-10-12

**Authors:** Menelaos Papakonstantinou, Paraskevi Chatzikomnitsa, Sophia Tsokkou, Areti Danai Gkaitatzi, Dimitrios Giakoustidis, Vasileios N. Papadopoulos, Alexandros Giakoustidis

**Affiliations:** First University Surgical Department, Papageorgiou General Hospital, Aristotle University of Thessaloniki, 541 24 Thessaloniki, Greecealexgiakoustidis@gmail.com (A.G.)

**Keywords:** sarcopenia, hepatopancreatobiliary surgery, skeletal muscle index, computed tomography, cut-off heterogeneity, methodological heterogeneity

## Abstract

**Background/Objectives**: Sarcopenia has emerged as an important prognostic factor in patients undergoing hepatopancreatobiliary (HPB) surgery. However, substantial heterogeneity in assessment methods and diagnostic thresholds limits comparability across studies. The aim of this review is to systematically characterize methodological variability in sarcopenia assessment within HPB surgical literature, quantify dispersion in reported cut-off values, and highlight key areas requiring standardization. **Methods**: A structured search of PubMed, Scopus, and Web of Science was conducted to identify studies evaluating sarcopenia in adult patients undergoing surgery for benign or malignant HPB diseases. The included studies assessed skeletal muscle mass with computed tomography (CT)-based measures, rather than sarcopenia as defined in contemporary consensus definitions. Data were extracted regarding imaging modality, anatomical landmarks, muscle indices, normalization strategies and diagnostic thresholds. Between-study variability in skeletal muscle index (SMI) thresholds was quantified using the coefficient of variation (CV). **Results**: Fifty-three studies comprising 11,613 patients met the inclusion criteria. Thirty-four studies assessed CT-defined low skeletal muscle mass using total skeletal muscle area at the third lumbar vertebral level normalized for height, whereas the remaining studies employed heterogeneous methodologies, including psoas-only measurements and alternative vertebral levels. Reported SMI cut-off values ranged from 36.2 to 53.5 cm^2^/m^2^ in males and 29.6 to 41.1 cm^2^/m^2^ in females. The CV for male and female cut-offs was 10.9% and 7.6%, respectively. Descriptively, lower CV values were observed in Western cohorts (male 6.5%; female 4.2%) than in Eastern cohorts (male 11.0%; female 9.0%); these values were not formally compared statistically. Considerable dispersion persisted irrespective of the threshold source. **Conclusions**: Despite the predominance of CT-derived L3 SMI assessment, marked methodological heterogeneity remains in HPB sarcopenia research. Variability in anatomical landmarks, normalization methods, and cut-off values limits reproducibility and constrains meta-analytic synthesis. Harmonization of reporting and threshold selection is essential to clarify the prognostic role of sarcopenia in HPB surgery.

## 1. Introduction

Sarcopenia is characterized by a progressive loss of skeletal muscle mass and strength, typically age-related, and is currently recognized as a potentially reversible muscle disease [1]. Systemic diseases, including malignancy, may accelerate muscle loss and exacerbate its clinical consequences. Body composition varies considerably among patients with cancer, and sarcopenia has been identified as a determinant of cancer cachexia severity. It is also associated with impaired functional status, reduced survival and increased chemotherapy toxicity [2,3]. The recognition that nutritional status and muscle mass are frequently compromised in oncologic patients has led to growing interest in the impact of sarcopenia on clinical outcomes, particularly postoperative results.

Several organizations have proposed modified versions of sarcopenia definitions, and an effort toward standardization began in 2010 with the first consensus of the European Working Group on Sarcopenia in Older People (EWGSOP), which defined sarcopenia as low muscle mass and either low muscle strength or impaired physical performance. The condition was further categorized as age-related, activity-related, disease-related, or nutrition-related. In 2019, EWGSOP revised the definition, distinguishing physical performance as an indicator of severity rather than a diagnostic criterion [1,4]. The increasing clinical interest culminated in the Global Leadership Initiative in Sarcopenia (GLIS) in 2024, which proposed a global conceptual definition. GLIS defined sarcopenia as the presence of reduced muscle mass and reduced muscle strength, without incorporating severity grading, and clarified that physical performance should be considered an outcome rather than a diagnostic component of sarcopenia [5].

Computed tomography (CT) is considered the “gold standard” for skeletal muscle mass assessment [4]. Several CT-based approaches have been proposed; total-body CT would provide the most accurate estimate of overall muscle mass, but its routine use is neither practical nor cost-effective. Therefore, surrogate measurements have been developed. The psoas muscle area has been widely utilized due to its simplicity; however, as a relatively small muscle group, it may not accurately reflect total skeletal muscle mass [6]. Increasing evidence supports the use of total muscle area at the level of the third lumbar vertebra (L3), which strongly correlates with whole-body muscle mass [3]. Cross-sectional muscle area does not account for body size; therefore, normalization strategies have been introduced. The skeletal muscle index (SMI), defined as the cross-sectional muscle area at L3 normalized to height squared (cm^2^/m^2^), is the most commonly used metric. Alternative indices, such as the psoas muscle index (PMI), may underestimate total muscle mass. The use of multiple indices contributes to methodological heterogeneity, limiting comparability across studies.

Cut-off thresholds for defining sarcopenia vary considerably. Although specific SMI cut-offs have been proposed, they are not consistently adopted [2,3]. The EWGSOP consensus provides cut-offs for grip strength, gait speed, and appendicular muscle mass, but not for CT-based SMI [4]. Similarly, the AWGS proposes cut-offs for dual-energy X-ray absorptiometry (DXA), bioelectrical impedance analysis (BIA), or calf circumference, but not for SMI [7]. Many studies derive their own thresholds or adopt previously published values, further increasing variability and limiting comparability.

Sarcopenia has gained clinical importance due to its association with adverse postoperative outcomes, including increased complications, prolonged hospital stay, and reduced survival following major abdominal surgery [8]. In hepatopancreatobiliary (HPB) surgery, evidence suggests increased morbidity and poorer survival; however, methodological heterogeneity and limited data hinder definitive conclusions [9]. Patients with HPB malignancies may be particularly vulnerable due to tumor-related metabolic derangements, reduced caloric intake and increased catabolism [10]. Despite growing interest, substantial variability in assessment methods and cut-off values persists, particularly in HPB surgery, where smaller cohorts further amplify methodological inconsistency [11].

The aim of this scoping review is to summarize the current literature regarding sarcopenia assessment methodology in patients undergoing surgery for benign or malignant HPB diseases. We evaluate the diagnostic approaches employed, quantify methodological heterogeneity, identify existing gaps, and propose directions toward a standardized reporting framework for future research.

## 2. Materials and Methods

### 2.1. Search Strategy

A comprehensive literature search was conducted in PubMed, Scopus, and Web of Science to identify studies evaluating sarcopenia in patients undergoing surgery for benign or malignant HPB diseases. The last search was performed in December 2025. The full electronic search strategy is presented in Supplementary Table S1. A total of 687 records were retrieved and imported into Rayyan (Qatar Computing Research Institute) for screening. Duplicate records were identified automatically and subsequently verified and removed manually by two independent reviewers (MP and ADG). Title, abstract and full-text screening was performed independently by the same two reviewers (MP and ADG) according to predefined inclusion and exclusion criteria.

**Figure 1:**
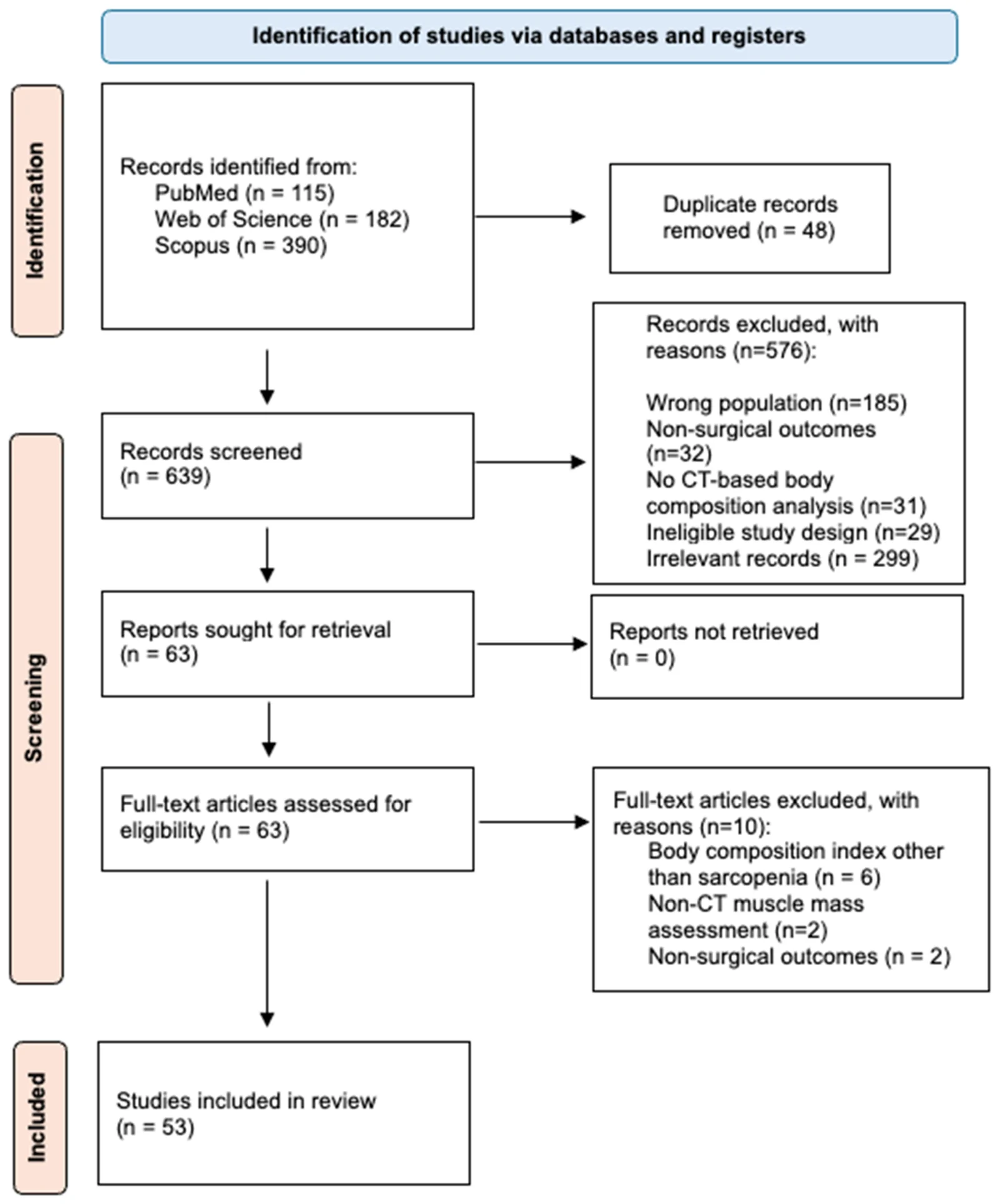
PRISMA flowchart.

After removing 48 duplicate and 576 irrelevant articles and records reporting non-surgical outcomes and non-CT-based body composition analysis in their abstracts, 63 studies were eligible for full-text assessment. Ten studies were excluded; six did not specifically evaluate sarcopenia, two did not include CT-based muscle mass assessment, and two did not report surgical outcomes. Ultimately, 53 studies met the eligibility criteria and were included in the final analysis. Discrepancies during any stage of the selection process were resolved through discussion with a senior reviewer (AG). The screening and selection process was performed according to the PRISMA extension for scoping reviews and is illustrated in Figure 1 [12]. A protocol was not pre-registered for this scoping review.

### 2.2. Inclusion and Exclusion Criteria

Eligible studies were original prospective or retrospective cohort studies published in English that evaluated sarcopenia using radiological methods in adult patients undergoing hepatic, pancreatic, or biliary surgery for benign or malignant conditions. Studies were included if they assessed CT-derived skeletal muscle mass using predefined cut-off thresholds, irrespective of whether additional functional measures were incorporated, since the former are frequently used as a surrogate for sarcopenia diagnosis in surgical literature.

Studies were excluded if they: (1) included non-surgical populations, (2) focused on transplantation, trauma or non-HPB procedures, (3) evaluated adiposity indices (such as visceral fat, intramuscular fat, or sarcopenic obesity) without defining sarcopenia, (4) did not include CT-based body composition analysis, (5) reported exclusively non-surgical outcomes (such as financial or resource utilization data), or (6) were reviews, meta-analyses, editorials, commentaries, letters to the editor, case-reports, or conference abstracts.

### 2.3. Data Extraction

Data were extracted from each eligible study using a predefined data collection sheet. Extracted variables included first author, year of publication, country of origin, journal, study design, sample size, type of surgery, body composition assessment method, imaging modality, software used for muscle segmentation, vertebral level selected for muscle area measurement (e.g., L3, L4), muscle groups included in the assessment, muscle area normalization index (e.g., SMI, PMI), normalization method, sex-specific cut-off thresholds, source of threshold (study-derived or literature-based) and primary and secondary outcomes (such as postoperative complications, survival, mortality, or length of stay).

### 2.4. Data Synthesis and Analysis

Included studies were summarized descriptively, with particular emphasis on methodological heterogeneity in sarcopenia assessment across HPB literature. Variables of interest included vertebral level selection, normalization method, and the index diagnostic thresholds. To quantify the variability in reported SMI cut-off values, the coefficient of variation (CV) was calculated separately for male and female thresholds using the formula:

CV = (Standard deviation of SMI cut-off values)/(Mean SMI cut-off) × 100%.

Sample standard deviation was used for all calculations. Identical thresholds reported across multiple studies were handled as repeated observations, to describe the realistic variability of thresholds as applied across the published literature. Studies reporting more than one sex-specific SMI threshold for the same population were not included in the CV analysis to avoid treating multiple alternative cut-offs derived from the same population as independent observations and increasing the contribution of the study in the estimated variability. Studies with missing values were excluded from the analysis. A sensitivity analysis based on unique numerical thresholds was performed to distinguish the distribution of distinct cut-offs from their frequency of use across the studies. For the descriptive characteristics of the included literature, the unit of analysis was the individual study, whereas for the CV analyses the unit of analysis was the cohort with an explicit sex-specific threshold; the two units are reported separately and are labeled explicitly throughout the Results. Studies were classified as Western (Europe and North America) or Eastern (Asia, including Western Asia, i.e., Turkey and Israel) according to the country of the reporting center, and where a single study reported separate Eastern and Western cohorts these cohorts were classified separately in the cohort-level analyses. When a study applied several published threshold sets, or body mass index (BMI)-stratified variants of a single criterion, the definition used for that study’s primary analysis was extracted (for BMI-stratified male thresholds, the value applicable to a BMI < 25 kg/m^2^), whereas studies that applied multiple alternative definitions without designating a primary one were excluded from the CV analysis. All extracted threshold values were re-checked against the primary publications during revision, and the corrected dataset is reported in Supplementary Tables S2 and S3.

A low CV indicates limited relative variability, reflecting clustering of the cut-off values around the mean, whereas a high CV indicates a substantial dispersion. Comparative analysis was restricted to SMI thresholds derived from total skeletal muscle area measured at the L3 vertebral level, as alternative vertebral levels or isolated muscle groups did not yield directly comparable indices. Subgroup analyses were performed based on the geographic region of study origin, the organ of interest (liver, pancreas, or biliary tree), and the source of threshold used (study-derived or literature-based). Based on the findings of the synthesis, we aim to propose a standardized reporting framework to enhance comparability and reproducibility in future HPB sarcopenia research.

Categorical variables were reported as frequencies and percentages, while continuous variables were reported as mean and standard deviation (SD) or median and range, depending on normal or non-normal distribution, respectively. All statistical analyses were performed using IBM SPSS Statistics (version 29.0.2.0).

## 3. Results

### 3.1. Studies That Assessed Total Muscle Area at the Level of L3

Thirty-four studies assessed CT-defined low skeletal muscle mass in a total of 8158 patients by measuring total skeletal muscle area at the L3 level [13,14,15,16,17,18,19,20,21,22,23,24,25,26,27,28,29,30,31,32,33,34,35,36,37,38,39,40,41,42,43,44,45,46]. The muscle groups included the psoas major, quadratus lumborum, erector spinae, multifidus, rectus abdominis, transversus abdominis, and the internal and external oblique muscles. Muscle area was semi-automatically quantified using dedicated software based on muscle density in Hounsfield Units (HU) as recognized on CT scans. In 23 studies, muscle density thresholds ranged from −29 to 150 HU, while four studies used −30 to 150 and one used −30 to 110. Six studies did not report the applied HU range. The SMI was calculated by normalizing the total muscle area (cm^2^) to the height squared (m^2^).

In this descriptive section the unit of analysis is the individual study (n = 34); the one study that reported separate Eastern and Western patient cohorts is counted once, according to the country of the reporting center. Most studies were retrospective (91.2%, n = 31) and 7 (20.6%) were multicenter. Twenty-one studies (61.8%) originated from Eastern countries—10 from China (29.4%), 8 from Japan (23.5%), one from Korea (2.9%) and one each from Taiwan and Israel—while 13 studies (38.2%) originated from Western countries (Italy n = 4, the Netherlands n = 3, France n = 3, Germany n = 2, Denmark n = 1). All included studies reported surgical outcomes, including postoperative complications and/or survival of patients with HPB diseases (Table 1). Across the 33 cohorts with an explicit sex-specific threshold, the SMI cut-off values ranged from 36.2 cm^2^/m^2^ to 53.5 cm^2^/m^2^ in males, and from 29.6 cm^2^/m^2^ to 41.1 cm^2^/m^2^ in females. Twenty-four cohorts applied literature-derived cut-offs while 9 generated cohort-specific thresholds using statistical methods such as receiver operating characteristic (ROC) analysis, percentile-based definitions, or deviations from cohort means (Table 2).

Beumer et al. reported separate Eastern and Western patient cohorts with distinct cut-off values, whereas Wu et al. applied both Western and Eastern diagnostic criteria to a single Taiwanese cohort [15,27]. Both studies are reported once in Table 1 and Table 2. Beumer et al. was excluded from the cut-off analysis because seven alternative SMI thresholds were applied to the same patients without designating a primary definition. For Wu et al., the Eastern criteria applicable to the Asian study population were extracted, so that study contributes a single cohort rather than two.

#### Primary CV Analysis

Thirty-three cohorts with explicit sex-specific cut-offs were included. To assess the relative variability in reported SMI thresholds, the CV was calculated separately for male and female cut-off values.

The CV was 10.9% for male cut-offs and 7.6% for female cut-offs, indicating substantial dispersion in the thresholds applied to patients of both sexes, which was somewhat greater for male thresholds. To explore potential sources of variability, subgroup analyses were performed based on geographic region, target organ, and threshold source.

### 3.2. Variability by Geographical Region

When stratified by region, 12 Western and 21 Eastern cohorts were available. Descriptively, lower CV values were observed in the Western cohorts (6.5% for male and 4.2% for female cut-offs) than in the Eastern cohorts (11.0% for male and 9.0% for female cut-offs). Confidence intervals for the CV were not calculated and no formal statistical comparison between regions was performed; this difference is therefore reported as a descriptive observation and should not be interpreted as evidence of a statistically significant difference in threshold homogeneity.

### 3.3. Variability by Target Organ

We assessed the difference in CV depending on the underlying HPB disease. In cohorts of liver diseases (n = 15), the CV was 10.4% for male cut-offs and 6.9% for female cut-offs. In pancreatic diseases (n = 12) the CV for the reported cut-offs was 12.2% and 8.8% for males and females, and in biliary diseases (n = 6) 10.8% and 3.1%, respectively. Variability differed between organs and sexes, and no single disease category demonstrated uniformly low dispersion across both sexes.

### 3.4. Variability by Threshold Source

When stratified by threshold origin, variability remained high irrespective of derivation method. In studies that assessed sarcopenia based on cohort-derived cut-offs, the CV was 10.0% for male cut-offs and 6.0% for female cut-offs, while in studies using literature-derived cut-offs, the CV was 11.3% for male and 8.2% for female cut-offs. Thus, adoption of previously published cut-offs did not consistently reduce inter-study variability.

#### Unique-Threshold Analysis

In the primary CV analysis, thresholds from all the included studies were used in the CV calculation. In this unique-threshold analysis we used each reported threshold once, even if the same threshold was used in more than one study. This distinction offered a better understanding of the distribution of unique cut-offs by not accounting frequency of use.

When each distinct numerical threshold was counted only once, the corresponding CV for males and females was 10.8% and 9.1%, respectively. Thus, the repeated use of identical thresholds resulted in only modest change in the estimated variability of the SMI cut-offs used in the literature.

The Coefficient of variation of the reported SMI cut-off values can be see in Table 3 and the complete dataset used to calculate the CV is available as Supplementary Material (Supplementary Tables S2 and S3).

### 3.5. Studies with Heterogeneous Assessment of CT-Defined Low Skeletal Muscle Mass

Nineteen studies employed alternative methodologies for the assessment of CT-defined low skeletal muscle mass in 3455 patients, using varying anatomical levels and muscle indices [47,48,49,50,51,52,53,54,55,56,57,58,59,60,61,62,63,64,65]. Measurement sites included the second (L2) (n = 1), third (L3) (n = 15) or fourth (L4) (n = 2) lumbar vertebral level, and one study measured across both L3 and L4 levels on preoperative CT imaging. Two of the studies assessed sarcopenia by measuring the total muscle area at L3 but applied alternative indices, including visceral fat area to total abdominal muscle area (VFA/TAMA), and skeletal muscle area (SMA) without height normalization. The most frequently reported alternative index was the psoas muscle index (PMI), calculated as bilateral psoas muscle area normalized to height squared of the patient. Additional reported indices included VFA/TAMA, skeletal muscle area (SMA), skeletal muscle index (SMI), lean psoas muscle area (LPMA), total psoas index (TPI), total psoas area index (TPAI), total psoas muscle area (TPA), and Hounsfield Unit Average Calculation (HUAC). Each index was accompanied by distinct diagnostic thresholds. When low skeletal muscle mass was defined using the PMI, the reported prevalence ranged from 6.4% to 73.8%. When assessed using VFA/TAMA, the reported prevalence reached 80.1% (Table 4). These studies were conducted in Western (n = 8; United States n = 4, Italy n = 2, Germany n = 1, Ukraine n = 1) and Eastern (n = 11; Japan n = 4, Turkey n = 3, China n = 1, Taiwan n = 2, Israel n = 1) countries. Given the diversity of anatomical levels, indices, normalization methods, and cut-off definitions, quantitative synthesis of threshold values was not feasible for this subgroup.

A comprehensive table, including details on patients’ characteristics and the exact muscle area measurement methodology used in each study, is shown as Supplementary Table S4 for further reference.

## 4. Discussion

In this review, we systematically evaluated the heterogeneity in reporting and diagnosing sarcopenia among patients undergoing surgery for HPB diseases. Our results demonstrated variability in both assessment methodology and diagnostic thresholds. An important conceptual consideration is the distinction between sarcopenia and CT-derived low skeletal muscle mass. Contemporary consensus definitions require both muscle mass and muscle strength assessment for sarcopenia diagnosis. However, most of the studies included in this review classified patients solely according to CT-derived skeletal muscle measurements. Therefore, the methodological heterogeneity reported in this review primarily reflects variation in the assessment and definition of CT-derived low skeletal muscle mass, which is frequently treated as a substitute for sarcopenia in the present surgical research. Even though CT-based assessment predominates in contemporary HPB literature, its implementation still lacks standardization. The cross-sectional total muscle area at the L3 level is the most commonly used to calculate the SMI; however, alternative indices, such as the PMI, PMA, LPMA, or VFA/TAMA, are also frequently employed.

The continued use of psoas muscle measurements is increasingly recognized as methodologically limited, given that psoas represents only a minor component of total skeletal muscle mass and may not accurately reflect body muscle depletion [6]. Furthermore, several studies have used different vertebral levels for muscle assessment, further compounding heterogeneity. This diversity in anatomical landmarks, muscle groups and normalization strategies compromises comparability and limits the validity of pooled analyses.

Beyond measurement method, variability in diagnostic cut-off values represents a major source of heterogeneity. Across studies, thresholds varied widely, even when the same index was applied. Many investigators derived cohort-specific cut-offs using receiver operating characteristic (ROC) analysis, often in small samples. These data-driven thresholds may optimize discrimination within a specific population but limit generalizability. Notably, even literature-derived cut-offs demonstrated high dispersion (CV 11.3% for male and 8.2% for female thresholds), reflecting the absence of universally accepted SMI thresholds. Many were originally developed in specific populations and later adopted broadly despite differences in ethnicity, disease type, or clinical setting.

Regional differences in dispersion were also apparent descriptively. Lower CV values were observed in Western than in Eastern cohorts; these values were not compared statistically and no confidence intervals were derived, so this observation is hypothesis-generating rather than confirmatory. The apparent heterogeneous dispersion of the reported cut-off thresholds may be affected by patient characteristics, such as disease-specific factors, or patient geographic distribution and race. Other contributing factors may include differences in CT protocols, contrast use, slice thickness, segmentation software and operator experience. These technical parameters were themselves poorly reported. Among the 34 studies that used the L3 SMI, none reported the reconstructed slice thickness used for body composition analysis, 27 (79.4%) did not state whether intravenous contrast had been administered or which contrast phase was measured, 19 (55.9%) did not specify whether segmentation was manual, semi-automated or automated, and 9 (26.5%) did not name the segmentation software. Because muscle attenuation and therefore measured muscle area depend on all of these parameters, the reported cut-off values are not strictly interchangeable even between studies that use the same index at the same vertebral level, and this reporting gap is the direct motivation for the framework proposed below.

Our analysis showed somewhat greater relative variability for male than for female cut-off values when all reported thresholds were analyzed (10.9% vs. 7.6%), and this difference narrowed when only unique thresholds were considered (10.8% vs. 9.1%). The reasons remain unclear but may relate to narrower muscle mass distribution or more consistent application of reference cut-off values. Nonetheless, dispersion persisted across both sexes, underscoring the need for harmonized threshold definitions.

Importantly, variability in SMI cut-off has direct clinical implications. Small shifts in thresholds may reclassify borderline patients, altering reported prevalence and associations with postoperative outcomes. This may affect risk stratification, eligibility for prehabilitation, and effect size estimates in survival analyses, ultimately introducing bias and compromising comparability across studies.

Previous studies have similarly highlighted this methodological inconsistency. Ramos-Bossini et al. reported marked differences in sarcopenia prevalence depending on the assessment index and applied threshold, with prevalence varying substantially even within comparable clinical contexts [66]. In pancreatic cancer cohorts, reported sarcopenia prevalence has ranged from 10% to 86% despite use of SMI-based definitions, depending on segmentation methodology and analytic approach [67,68]. Such variation cannot be attributed solely to population differences and likely reflects inconsistencies in measurement technique, software algorithms, and operator-dependent segmentation. Contrast administration may further influence HU-based muscle delineation [69]. Moreover, classification based on categorical cut-offs may yield different prognostic associations compared to analysis of continuous SMI values. Continuous modeling has been suggested to provide superior predictive accuracy in certain surgical settings [69].

Collectively, these findings illustrate that methodological variability directly influences both sarcopenia prevalence and its prognostic interpretation. Without standardized acquisition parameters, segmentation protocols, normalization strategies, and threshold definitions, comparability across studies remains limited.

The variability in sarcopenia assessment methods impedes translation into clinical practice. CT scans, BIA, DXA, muscle radiodensity attenuation, and ultrasonography are only some of the methods that have been used to measure muscle mass [69]. But for sarcopenia to serve as a reliable risk stratification tool in HPB surgery, the diagnostic approach must be reproducible, widely accessible, cost-effective, and minimally burdensome for patients. Although CT imaging fulfills many of these criteria in oncologic surgery, uniform guidance regarding vertebral level, muscle groups, normalization method, and threshold selection remains lacking. Until such consensus is established, the true prognostic value of sarcopenia in HPB surgery cannot be fully delineated.

Beyond muscle quantity, emerging evidence underscores the importance of muscle quality [9]. Myosteatosis, characterized by increased intramuscular adipose tissue content (IMAC) or reduced muscle radiodensity attenuation (MRA), has been independently associated with postoperative complications and survival outcomes in HPB surgery [58,69]. Visceral adiposity has also been shown to contribute independently to adverse outcomes, particularly in hepatocellular carcinoma [70]. These findings suggest that composite models integrating both sarcopenia and visceral adiposity may enhance risk prediction for patients with HPB diseases. Future research should therefore consider multidimensional body composition assessment rather than isolated muscle mass metrics.

The 2024 GLIS consensus represents an important step toward harmonization, offering an operational definition for clinical research [5]. However, further clarification is required regarding imaging modality, vertebral level selection, segmentation standards, normalization approach (height versus BMI adjustment), and sex-specific thresholds [7,50,57]. Operator training, software standardization, and quality control measures should be incorporated into future guideline development. Artificial intelligence (AI)-based segmentation tools may further reduce operator-dependent variability and enhance reproducibility, as preliminary data in lung cancer suggest [71].

Based on the methodological variability identified in this review, we developed a practical reporting framework for CT-based assessment of skeletal muscle mass and sarcopenia in HPB surgery (Table 5). The proposed checklist includes 17 items in 8 domains of standardized reporting of muscle assessment. Specifically, it includes proposals for standardized reporting of CT acquisition parameters, anatomical landmarks and muscle groups, segmentation methodology, normalization strategy, threshold selection and outcomes. This framework is intended to improve transparency and reproducibility of future research and not substitute consensus-based guidelines or diagnostic algorithms.

## 5. Strengths and Limitations

The strengths of this study include a comprehensive review of the available literature and the construction of a structured dataset enabling quantitative assessment of the cut-off variability. By focusing on CT-based assessments and calculating CV, we provide objective measurement of methodological dispersion across studies. In the primary CV analysis, each SMI threshold was treated as an observation. Consequently, thresholds repeatedly adopted from the literature contributed proportionally to their frequency of use and the resulting CV reflected both the dispersion of reported thresholds and their frequency of application. This approach was considered appropriate for describing methodological heterogeneity in the published literature, but it does not represent a distribution of independent threshold values. To address this issue, we additionally evaluated the distribution of unique numerical thresholds, which reflected the dispersion of distinct proposed cut-offs. This analysis produced comparable CV values for male (10.8% vs. 10.9%) and female thresholds (9.1% vs. 7.6%), suggesting that the heterogeneity observed is not driven by the repeated use of the same cut-offs. Finally, we proposed a standardized assessment of muscle mass and sarcopenia in HPB studies in the form of a reporting checklist.

Several limitations of this review warrant consideration. Most included studies were retrospective observational cohorts, subject to inherent risks of selection and confounding bias, and variable data quality. Sarcopenia assessment relied exclusively on CT-derived muscle quantity; therefore, our findings should not be interpreted as representing the multidimensional assessment of sarcopenia as proposed by contemporary consensus definitions. In addition, non-CT based methods were excluded, potentially limiting the breadth of evaluated muscle mass assessment strategies. However, CT remains the predominant modality for sarcopenia assessment in surgical populations, and our focus reflects the current clinical practice. Finally, variability in reporting quality across studies may have influenced data extraction accuracy.

## 6. Clinical Implications

CT-defined low skeletal muscle mass has been associated with increased postoperative complications and reduced survival in HPB surgery, although methodological heterogeneity currently limits direct comparison of assessment thresholds and formal synthesis of the available evidence [11]. Reliable and reproducible assessment is therefore essential before CT-based assessment of skeletal muscle mass can be incorporated confidently into clinical risk stratification or used to identify patients who may benefit from targeted prehabilitation. Standardized assessment and reporting may ultimately facilitate more consistent patient selection, comparison of outcomes and evaluation of muscle-targeted interventions in HPB surgery.

## 7. Conclusions

CT-defined low skeletal muscle mass is increasingly used as a prognostic marker in HPB surgery, yet in most studies it is assessed from muscle quantity alone and therefore does not correspond to sarcopenia as defined by current consensus criteria, which also require reduced muscle strength. Although total skeletal muscle area at L3 normalized to height squared is the most widely applied measure, the included studies differed in vertebral level, muscle groups, normalization method and diagnostic threshold, and frequently omitted essential details of image acquisition and segmentation. Even among studies using the same index, sex-specific cut-offs varied considerably, and adoption of previously published thresholds did not reduce this dispersion. Consequently, prevalence estimates and outcome associations are not directly comparable across studies, which limits reproducibility and precludes robust quantitative synthesis. Future studies should apply a standardized CT protocol and segmentation approach, justify and report the exact sex-specific thresholds used, present SMI as a continuous variable alongside categorical classification, and incorporate muscle strength assessment where feasible. Consistent reporting, as outlined in the proposed checklist, is a prerequisite for multicenter validation of thresholds and for establishing the true prognostic value of sarcopenia in patients undergoing HPB surgery.

## Figures and Tables

**Table 1:** Prevalence of CT-defined low skeletal muscle mass in studies assessing muscle mass with the SMI.

Author	Country	Region	Study Design	Outcome	Low Muscle Mass (%)
Berardi	Italy	Western	Prospective	Complications	61
Beumer	Nether	Western & Eastern	Retrospective	Survival	NS
Coelen	Nether	Western	Retrospective	Complications	42
El Amrani	France	Western	Retrospective	Complications	47
Meister	German	Western	Retrospective	Complications	54
Pecchi	Italy	Western	Retrospective	Survival	NS
Pessia liver	Italy	Western	Retrospective	Survival	64.9
Pessia pancreas	Italy	Western	Retrospective	Survival	47.1
Runkel	German	Western	Retrospective	Complications	36.2
Seror	France	Western	Retrospective	Complications	42
Van Vledder	Nether	Western	Retrospective	Survival	19.4
Voron	France	Western	Retrospective	Survival	45.9
Wielsoe	Denmar	Western	Prospective	Complications	7
Bo	China	Eastern	Prospective	Complications	27.5
Cai	China	Eastern	Retrospective	POPF	36.4
Fukuhara	Japan	Eastern	Retrospective	Complications	14.2
Harimoto	Japan	Eastern	Retrospective	Survival	40.3
Hayashi	Japan	Eastern	Retrospective	Survival	35
Higashi	Japan	Eastern	Retrospective	Complications	24
Li 2021 [33]	China	Eastern	Retrospective	Survival	61.1
Li 2024 [34]	China	Eastern	Retrospective	Complications	61.9
Lv	China	Eastern	Retrospective	Survival	57.3
Nishida	Japan	Eastern	Retrospective	POPF	49.6
Omiya	Japan	Eastern	Retrospective	Survival	NS
Qu	China	Eastern	Retrospective	Survival	NS
Rom	Israel	Eastern	Retrospective	Survival	24
Ryu	Korea	Eastern	Retrospective	POPF	45.9
Shen	China	Eastern	Retrospective	Survival	61.6
Sui	Japan	Eastern	Retrospective	Survival	24.6
Wu 2023 [43]	China	Eastern	Retrospective	Textbook outcomes	35.9
Wu 2019 [27]	Taiwan	Eastern	Retrospective	Survival	10.9
Yasuta	Japan	Eastern	Retrospective	Survival	46
Zhao	China	Eastern	Retrospective	Survival	63.6
Zhou	China	Eastern	Retrospective	Survival	49.3

POPF, postoperative pancreatic fistula; NS, not specified.

**Table 2:** Sex-specific SMI cut-off values and threshold sources.

Author	Male Cut-Off	Female Cut-Off	Threshold Source
Berardi	53.5	40.8	Own
Beumer	*	*	Literature
Coelen	46.8	39.1	Own
El Amrani	52.4	38.5	Literature
Meister	50	39	Literature
Pecchi	52.4	38.5	Literature
Pessia liver	52.4	38.5	Literature
Pessia pancreas	52.4	38.5	Literature
Runkel	52.4	38.5	Literature
Seror	52	38	Literature
Van Vledder	43.75	41.1	Own
Voron	52.4	38.9	Literature
Wielsoe	45.4	34.4	Literature
Bo	51.1	37.1	Own
Cai	42.2	33.9	Literature
Fukuhara	42	38	Literature
Harimoto	43.75	41.1	Literature
Hayashi	52.4	38.9	Literature
Higashi	43.2	35.3	Own
Li 2021 [33]	42.6	37.8	Own
Li 2024 [34]	53.5	39.9	Own
Lv	43	41	Literature
Nishida	43	41	Literature
Omiya	42	38	Literature
Qu	42	38	Literature
Rom	44.35	34.82	Own
Ryu	50.18	38.63	Literature
Shen	52.4	38.5	Literature
Sui	40.5	33.5	Literature
Wu 2023 [43]	40.89	30.71	Literature
Wu 2019 [27]	36.2	29.6	Literature
Yasuta	43	41	Literature
Zhao	53.5	39.9	Own
Zhou	43.75	41.1	Literature

SMI: skeletal muscle index. * Beumer et al. compared the effect of 7 different SMI thresholds on clinical outcomes.

**Table 3:** Coefficient of variation of the reported SMI cut-off values.

		Male Cut-Off CV (%)	Female Cut-Off Cv (%)
	Overall	10.9	7.6
Different region	Western cohorts	6.5	4.2
	Eastern cohort	11.0	9.0
Different disease	Liver disease	10.4	6.9
	Pancreatic disease	12.2	8.8
	Biliary disease	10.8	3.1
Different source	Own threshold	10.0	6.0
	Literature threshold	11.3	8.2

CV, coefficient of variation.

**Table 4:** Miscellaneous methods for the assessment of CT-defined low skeletal muscle mass.

Author	Muscles Measured	Vertebral Level	Index Used	Male Cut-Off	Female Cut-Off	Threshold Source	Low Muscle Mass (%)
Benjamin	Psoas	L3	NS	NS	NS	Own cohort	NS
Centonze	Psoas	L3	HUAC	16.37	14.21	Literature	32.72
Deng	Psoas	L3	SMI	8.6	6.04	Own cohort	73.8
Guarneri	Total muscle area	L3	VFA/TAMA	NS	NS	Literature	80.1
Kilinc	Total muscle area	L3	SMA	113	87	Own cohort	48.3
Kopchak	Psoas	L3	HUAC	18.8	20.3	Own cohort	43.4
Lee	Psoas	L2	PMI	2.305	1.503	Own cohort	6.4
Linder	Psoas and paraspinal	L3–L4	SMI	NS	NS	Literature	NS
Liu	Psoas and paraspinal	L4	HUAC	22	22	Own cohort	26.4
Mercan	Psoas	L3	TPI	4.23	2.73	Own cohort	NS
Nauheim	Psoas	L3	PMI	NS	NS	Own cohort	NS
Okumura	Psoas	L3	PMI	6.15	3.98	Own cohort	34.3
Onesti	Psoas	L4	LPMA	NS	NS	Literature	NS
Otsuji	Psoas	L3	TPA	NS	NS	Own cohort	33.2
Shintakuya	Psoas	L3	PMI	Lowest quartile	Lowest quartile	Literature	NS
Sur	Psoas	L3	TPAI	NS	NS	NS	NS
Tankel	Psoas	L3	TPA	83.41	65.28	Literature	26.2
Tazeoglu	Psoas	L3	PMI	5.3	3.6	Literature	46.4
Umetsu	Psoas	L3	PMI	5.93	3.54	Own cohort	73.8

L3, third lumbar vertebral level; NS, not specified; HUAC, Hounsfield unit average calculation; SMI, skeletal muscle index; VFA/TAMA, visceral fat area to total abdominal muscle area; SMA, skeletal muscle area; PMI, psoas muscle index; L2, second lumbar vertebral level; L4, fourth lumbar vertebral level; TPI, total psoas index; LPMA, lean psoas muscle area; TPA, total psoas muscle area; TPAI, total psoas area index.

**Table 5:** Proposed reporting checklist for CT-based assessment of skeletal muscle mass and sarcopenia in HPB surgery.

Domain	Recommended Reporting Item	Minimum Recommended Information to Report
Patient assessment	Clinical/functional assessment	Report whether muscle mass and muscle strength and/or physical performance were assessed. Specify methods used (CT-based assessment, DXA, BIA, handgrip strength, gait speed, etc.), protocol, units, and cut-offs where applicable.
Sarcopenia definition	Diagnostic framework	State explicitly whether muscle strength was assessed in addition to muscle mass. If not, acknowledge that the study reports CT-defined low skeletal muscle mass rather than sarcopenia as defined by the GLIS consensus.
CT parameters	Timing of CT	Specify the interval between CT acquisition and surgery or the defined clinical time point used for assessment.
	CT phase	Report whether CT was performed with or without contrast, and if applicable, the phase used for muscle assessment (e.g., arterial, portal, delayed).
	Slice thickness	Report the reconstructed slice thickness used for body composition analysis.
	HU range	Report the HU range used to identify skeletal muscle.
Anatomical landmark selection	Vertebral level	Specify the vertebral level used for measurement (e.g., L3).
	Muscle groups	Specify all muscles included in the measurement.
Segmentation	Software	Report the software and version used for segmentation.
	Method	Specify whether segmentation was manual, semi-automated, or automated/AI-based.
	Manual correction	If automated or semiautomated segmentation method was used, report whether manual correction was performed, and describe the correction procedure and who performed it.
	Observer reliability	Report the number and expertise of observers and, where applicable, intra- and/or interobserver reliability measures.
Quantification	Normalization method	Specify how skeletal muscle area was normalized (e.g., height squared, BMI), and provide the resulting index (e.g., SMI).
Threshold definition	Threshold source	State whether the threshold was study-derived, consensus-based, or adopted from the previous literature and cite the original source.
	Threshold selection	Provide the rationale for selecting the threshold and report the exact sex-specific and population-specific values.
Outcome reporting	Continuous values	Report mean ± SD or median (IQR) of the index used (e.g., SMI) for the whole cohort and subgroups.
	Categorical sarcopenia status	Report the proportion of patients classified as sarcopenic (prevalence) using the applied threshold and definition.

CT, computed tomography; HPB, hepatopancreatobiliary; DXA, dual-energy X-ray absorptiometry; BIA, bioelectrical impedance analysis; HU, Hounsfield units; AI, artificial intelligence; BMI, body mass index; SMI, skeletal muscle index; SD, standard deviation; IQR, interquartile range.

## Data Availability

The data that support the findings of this study are available on request from the corresponding authors.

## Supplementary Materials

The following supporting information can be downloaded at: https://www.mdpi.com/article/10.3390/jcm15207856/s1, Table S1. Search strings used in PubMed, Web of Science and Scopus. Table S2. Complete dataset used for the coefficient of variation analyses. Table S3. Coefficient of variation calculation. Table S4. Complete study characteristics.
