# Supplementary material for: Heterogeneity in CT-Based Skeletal Muscle Mass Assessment and Sarcopenia Diagnosis Undermines Comparability in Hepatopancreatobiliary Surgery: A Scoping Review
Source: J Clin Med. 2026 Oct 12;15(20):7856. doi: 10.3390/jcm15207856 (PMC13705426; doi:10.3390/jcm15207856)
Supplement: Supplementary file 1 [file jcm-15-07856-s001.zip › jcm-4476112-supplementary.pdf]

Supplementary Table S1. Search strings used in PubMed, Web of Science and Scopus.

Database	Search string	Filters	Date searched
**Pubmed**	("CT"[tiab] OR "computed tomography"[tiab] OR "skeletal muscle"[tiab]) AND ("Sarcopenia"[Mesh] OR sarcopenia[tiab] OR "muscle wasting"[tiab] OR "low muscle mass"[tiab] OR "low skeletal muscle"[tiab] OR "skeletal muscle index"[tiab] OR SMI[tiab] OR "muscle mass"[tiab] OR "reduced muscle mass"[tiab] OR sarcopenic[tiab]) AND ("Hepatectomy"[Mesh] OR "Pancreatectomy"[Mesh] OR "Bile Duct Neoplasms"[Mesh] OR hepatobiliary[tiab] OR hepatopancreatobiliary[tiab] OR HPB[tiab] OR liver[tiab] OR hepatic[tiab] OR hepatectomy[tiab] OR "liver resection"[tiab] OR "liver resection"[tiab] OR pancreas[tiab] OR pancreatic[tiab] OR pancreatectomy[tiab] OR "pancreaticoduodenectomy"[tiab] OR "Whipple"[tiab] OR biliary[tiab] OR bile[tiab] OR cholangiocarcinoma[tiab] OR cholangio*[tiab] OR "bile duct"[tiab] OR "biliary tract"[tiab]) AND ("Postoperative Complications"[Mesh] OR "Treatment Outcome"[Mesh] OR postoperative[tiab] OR "post-operative"[tiab] OR complication*[tiab] OR morbidity[tiab] OR mortality[tiab] OR survival[tiab] OR "length of stay"[tiab] OR LOS[tiab] OR recurrence[tiab] OR "readmission"[tiab] OR "time to adjuvant"[tiab] OR "disease-free survival"[tiab] OR "overall survival"[tiab])	Clinical Study, Clinical Trial, Comparative Study, Controlled Clinical Trial, Multicenter Study, Observational Study, Randomized Controlled Trial	8/12/2025
**Web of Science**	((sarcopenia OR "muscle wasting" OR "low muscle mass" OR "skeletal muscle index" OR SMI OR "reduced muscle mass" OR sarcopenic) AND (hepatobiliary OR hepatopancreatobiliary OR HPB OR liver OR hepatic OR hepatectomy OR "liver resection" OR pancreas OR pancreatic OR pancreatectomy OR "pancreaticoduodenectomy" OR Whipple OR biliary OR cholangio* OR "bile duct" OR "biliary tract") AND (postoperative OR "post-operative" OR complication* OR morbidity OR mortality OR survival	Surgery, Oncology, Gastroenterology Hepatology, English Language	8/12/2025

	OR "length of stay" OR LOS OR recurrence OR readmission OR "disease-free survival" OR "overall survival"))	
**Scopus**	(sarcopenia OR "low muscle mass" OR "skeletal muscle index" OR SMI) AND ("computed tomography" OR CT OR "skeletal muscle area" OR "cross-sectional muscle area") AND (liver OR hepatic OR hepatectomy OR "liver resection" OR pancreas OR pancreatic OR pancreatectomy OR "pancreaticoduodenectomy" OR Whipple OR biliary OR cholangiocarcinoma OR cholangio* OR hepatobiliary OR hepatopancreatobiliary) AND (surgery OR operation OR resection OR hepatectomy OR pancreatectomy OR pancreaticoduodenectomy) AND (outcome* OR complication* OR morbidity OR mortality OR survival) AND NOT (review OR "systematic review" OR "meta-analysis") AND (LIMIT-TO (DOCTYPE , "ar")) AND (LIMIT-TO (LANGUAGE , "English"))	8/12/2025

Supplementary Table S2. Complete dataset used for the coefficient of variation analyses.

Author	Male cut-off	Female cut-off	Region	Organ	Threshold source
**Berardi**	53.5	40.8	Western	Liver	Own
**Beumer**	*	*	Western	Liver	Literature
**Coelen**	46.8	39.1	Western	Biliary	Own
**El Amrani**	52.4	38.5	Western	Pancreas	Literature
**Meister**	50	39	Western	Liver	Literature
**Pecchi**	52.4	38.5	Western	Pancreas	Literature
**Pessia liver**	52.4	38.5	Western	Liver	Literature
**Pessia pancreas**	52.4	38.5	Western	Pancreas	Literature
**Runkel**	52.4	38.5	Western	Liver	Literature
**Seror**	52	38	Western	Liver	Literature
**Van Vledder**	43.75	41.1	Western	Liver	Own
**Voron**	52.4	38.9	Western	Liver	Literature
**Wielsoe**	45.4	34.4	Western	Pancreas	Literature
**Bo**	51.1	37.1	Eastern	Liver	Own
**Cai**	42.2	33.9	Eastern	Pancreas	Literature
**Fukuhara**	42	38	Eastern	Liver	Literature
**Harimoto**	43.75	41.1	Eastern	Liver	Literature
**Hayashi**	52.4	38.9	Eastern	Liver	Literature
**Higashi**	43.2	35.3	Eastern	Liver	Own
**Li 2021**	42.6	37.8	Eastern	Biliary	Own

**Li 2024**	53.5	39.9	Eastern	Biliary	Own
**Lv**	43	41	Eastern	Liver	Literature
**Nishida**	43	41	Eastern	Pancreas	Literature
**Omiya**	42	38	Eastern	Liver	Literature
**Qu**	42	38	Eastern	Pancreas	Literature
**Rom**	44.35	34.82	Eastern	Pancreas	Own
**Ryu**	50.18	38.63	Eastern	Pancreas	Literature
**Shen**	52.4	38.5	Eastern	Pancreas	Literature
**Sui**	40.5	33.5	Eastern	Pancreas	Literature
**Wu 2023**	40.89	30.71	Eastern	Liver	Literature
**Wu 2019**	36.2	29.6	Eastern	Pancreas	Literature
**Yasuta**	43	41	Eastern	Biliary	Literature
**Zhao**	53.5	39.9	Eastern	Biliary	Own
**Zhou**	43.75	41.1	Eastern	Biliary	Literature

Supplementary Table S3. Coefficient of variation calculation.

		SD	Mean	CV
**Overall (n=33)**	Male cut-off	5.12	47.01	10.9%
	Female cut-off	2.90	37.93	7.6%
**Eastern (n=21)**	Male cut-off	4.96	45.02	11.0%
	Female cut-off	3.38	37.51	9.0%
**Western (n=12)**	Male cut-off	3.28	50.49	6.5%
	Female cut-off	1.64	38.65	4.2%
**Liver (n=15)**	Male cut-off	4.94	47.65	10.4%
	Female cut-off	2.66	38.33	6.9%
**Pancreas (n=12)**	Male cut-off	5.64	46.12	12.2%
	Female cut-off	3.21	36.49	8.8%
**Biliary (n=6)**	Male cut-off	5.10	47.19	10.8%
	Female cut-off	1.24	39.80	3.1%
**Own threshold (n=9)**	Male cut-off	4.81	48.03	10.0%
	Female cut-off	2.31	38.42	6.0%
**Literature threshold (n=24)**	Male cut-off	5.28	46.63	11.3%
	Female cut-off	3.11	37.74	8.2%
**Unique thresholds (n=18)**	Male cut-off	4.91	45.56	10.8%
**Unique thresholds (n=19)**	Female cut-off	3.37	36.95	9.1%

Supplementary Table S4. Complete study characteristics.

Benjamin et al.	2017	134	pancreatic cancer	Pancreatectomy	NR	precontrast	NR	L3	Psoas	NR	NR	Manual	height squared	TPAI	own cohort	No	No	No	NR
**Berardi et al.**	2020	234	HCC, CCC, GBC, CRLM, nonCRLM	Hepatectomy	1 month	NR	NR	L3	total muscle area	-29 to 150	SliceOMatic	Manual	height squared	SMI	own cohort	Yes	Yes	-	None
**Beumer et al.**	2023	553	HCC	Hepatectomy	3 months	NR	NR	L3	psoas, erector spinae, quadratus lumborum, multifidus, transversus abdominis, external and	-30 to 150	FatSeg v4	Manual	height squared	SMI	Literature	No	No	No	Yes

									internal oblique and rectus abdominis										
**Bo et al.**	2023	120	benign liver disease	Hepatectomy	1 month	NR	NR	L3	total muscle area	-29 to 150	ImajeJ	NR	height squared	SMI	own cohort	Yes	Yes	-	Yes
**Cai et al.**	2023	129	periampullary neoplasms	Pancreatectomy	1 month	NR	NR	L3	total muscle area	-29 to 150	3D slicer	NR	height squared	SMI	Literature	No	No	No	None
**Centonze et al.**	2020	110	benign or malignant pancreatic disease	Pancreatectomy	NR	NR	NR	L3	Psoas	NR	NR	NR	HU report	HUAC	Literature	No	No	No	None
**Coelen et al.**	2015	100	perihilar cholangiocarcinoma	Hepatectomy	NR	NR	NR	L3	psoas, erector spinae	-30 to	OsiriX	NR	height squared	SMI	own cohort	No	No	Yes	NR

									, quadratus lumborum, multifidus, transversus abdominis, external and internal oblique and rectus abdominis	110									
**Deng et al.**	2021	121	ICC	Hepatectomy	1 month	non-contrast	NR	L3	Psoas	NR	NR	NR	height squared	SMI	own cohort	No	No	No	Yes
**El Amrani et al.**	2018	107	benign or malignant pancreatic disease	Pancreatectomy	NR	NR	NR	L3	psoas, erector spinae ,	-29 to 1	NR	Semiautomatic	height squared	SMI	Literature	No	No	No	NR

									quadratus lumborum, multifidus, transversus abdominis, external and internal oblique and rectus abdominis	50									
**Fukuhara et al.**	2024	253	HCC	Hepatectomy	1 month	NR	NR	L3	total muscle area	-29 to 150	Synapse Vincent	NR	height squared	SMI	Literature	Yes	Yes	-	Yes
**Guarneri et al.**	2024	371	pancreatic cancer	Pancreatectomy	1 month	NR	NR	L3	total muscle area	NR	SliceOMatic	NR	BMI	VFA/TAMA	Literature	No	No	No	None

**Harimoto et al.**	2013	186	HCC	Hepatectomy	NR	NR	NR	L3	psoas, erector spinae, quadratus lumborum, multifidus, transversus abdominis, external and internal oblique and rectus abdominis	-29 to 150	NR	Manual	height squared	SMI	Literature	No	No	No	NR
**Hayashi et al.**	2023	303	HCC	Hepatectomy	3 weeks	NR	NR	L3	total muscle area	-29 to 1	Synapse Vincent	NR	height squared	SMI	Literature	No	No	No	NR

										50									
**Higashi et al.**	2016	50	HCC	Hepatectomy	NR	NR	NR	L3	psoas, erector spinae, quadratus lumborum, multifidus, transversus abdominis, external and internal oblique and rectus abdominis	-29 to 150	Synapse Vincent	NR	height squared	SMI	own cohort	No	No	No	NR
**Kilinc-**	2023	89	periampullary	Pancreatectomy	NR	NR	NR	L3	psoas, erector	NR	NR	NR	-	SMA	own cohort	No	No	No	None

**Tuncer et al.**			malignant tumors						spinae, quadratus lumborum, multifidus, transversus abdominis, external and internal oblique and rectus abdominis										
**Kopchak et al.**	2021	152	pancreatic and periampullary cancer	Pancreatectomy or Appleby	1 month	NR	NR	L3	psoas	-30 to 110	NR	Semiautomatic	-	HUAC	own cohort	No	No	No	NR

**Lee et al.**	2024	450	stage I/II HCC	Hepatectomy	1 month	non-contrast	5	L2	psoas	-30 to 150	Matlab	Semiautomatic	height squared	PMI	own cohort	No	No	No	Yes
**Li et al.**	2021	460	ICC	Hepatectomy	NR	non-contrast	NR	L3	total muscle area	NR	Mimics	NR	height squared	SMI	own cohort	No	No	No	Yes
**Li et al. (2024)**	2024	260	ICC	Hepatectomy	2 months	NR	NR	L3	total muscle area	-29 to 150	NR	NR	height squared	SMI	own cohort	No	No	No	NR
**Linder et al.**	2019	139	periampullary malignant tumors	Pancreatectomy	2.5 months	venous	3 or 5	L3-L4	psoas and paraspinal muscles	-30 to 150	IDL	Manual	height squared	SMI	Literature	No	No	Yes	None
**Liu et al.**	2020	182	CRLM	Hepatectomy	1 month	NR	NR	L4	bilateral paraspinal,	-29 to	OsiriX	NR	-	HUAC	own cohort	No	No	No	NR

								psoas and abdominal wall	150									
**Lv et al.**	2019	539	CRLM	Hepatectomy	12 days	NR	NR	L3	psoas, erector spinae, quadratus lumborum, multifidus, transversus abdominis, external and internal oblique and rectus abdominis	-29 to 150	NR	NR	height squared	SMI	Literature	No	No	Yes	NR

**Meister et al.**	2022	100	HCC	Hepatectomy	3 months	NR	NR	L3	total muscle area	NR	3D slicer	Semiautomatic	height squared	SMI	Literature	No	No	No	Yes
**Mercan et al.**	2020	120	pancreatic cancer	Pancreatectomy	NR	NR	NR	L3	psoas	NR	NR	NR	height squared	TPI	own cohort	No	No	No	None
**Nauheim et al.**	2022	333	benign or malignant pancreatic disease	Pancreatectomy	45 days	NR	NR	L3	psoas	NR	IntelliSpace PACS	NR	BMI	PMI	own cohort	No	No	No	NR
**Nishida et al.**	2016	266	benign or malignant pancreatic disease	Pancreatectomy	NR	NR	NR	L3	total muscle area	-29 to 150	Synapse Vincent	NR	height squared	SMI	Literature	No	No	No	NR
**Okumura et al.**	2015	207	extrahepatic biliary cancer	Pancreatectomy or hepatectomy	1 month	NR	7	L3	psoas	NR	Aquarius	Manual	height squared	PMI	own cohort	No	No	No	NR
**Omiya et al.**	2021	93	HCC	Hepatectomy	NR	NR	NR	L3	total muscle area	-29 to 1	Ziostation	Automatic	height squared	SMI	Literature	No	No	No	NR

										50									
**Onesti et al.**	2016	270	benign or malignant pancreatic disease	Pancreatectomy	NR	NR	NR	L4	psoas	NR	NR	NR	-	LPMA	Literature	No	No	No	NR
**Otsuji et al.**	2017	256	benign or malignant liver disease	Hepatectomy	1 month	NR	NR	L3	psoas	NR	NR	Semiautomatic	height squared	TPA	own cohort	No	No	No	NR
**Pecchi et al.**	2024	88	pancreatic cancer	Pancreatectomy	NR	non-contrast	NR	L3	psoas, erector spinae, quadratus lumborum, multifidus, transversus abdominis, external and internal	-29 to 150	NR	Semiautomatic	height squared	SMI	Literature	No	No	No	None

									oblique and rectus abdominis								
**Pessia et al. (liver)**	2021	74	CRLM	Hepatectomy	NR	NR	NR	L3	total muscle area	NR	NR	Semiautomatic	height squared	SMI	Literature	No	No	No	NR
**Pessia et al. (panc)**	2021	68	pancreatic cancer	Pancreatectomy	NR	NR	NR	L3	psoas, erector spinae, quadratus lumborum, multifidus, transversus abdominis, external and internal obliqu	-29 to 150	NR	Semiautomatic	height squared	SMI	Literature	No	No	No	NR

									e and rectus abdominis										
**Qu et al.**	2024	162	pancreatic cancer	Pancreatectomy	NR	non-contrast	NR	L3	psoas, erector spinae, quadratus lumborum, multifidus, transversus abdominis, external and internal oblique and rectus abdominis	-29 to 150	SliceOMatic	NR	height squared	SMI	Literature	No	No	No	NR

**Rom et al.**	2022	111	pancreatic cancer	Pancreatectomy	180 days	non-contrast	NR	L3	psoas, erector spinae, quadratus lumborum, multifidus, transversus abdominis, external and internal oblique and rectus abdominis	-30 to 150	MeVis Lab	NR	height squared	SMI	own cohort	No	No	No	None
**Runkel et al.**	2021	94	CRLM	Hepatectomy	3 months	NR	NR	L3	total muscle area	-29 to 1	Aquarius	Semiautomatic	height squared	SMI	Literature	No	No	No	Yes

										50									
**Ryu et al.**	2020	548	pancreatic cancer	Pancreatectomy	NR	portal	NR	L3	total muscle area	NR	Matlab	NR	height squared	SMI	Literature	No	No	Yes	None
**Seror et al.**	2021	110	HCC	Hepatectomy	60 days	NR	NR	L3	total muscle area	NR	Horos	NR	height squared	SMI	Literature	No	No	No	None
**Shen et al.**	2023	614	pancreatic cancer	Pancreatectomy	1 week	NR	NR	L3	total muscle area	-29 to 150	NR	Semiautomatic	height squared	SMI	Literature	No	No	Yes	Yes
**Shintakuya et al.**	2019	112	pancreatic cancer	Pancreatectomy	NR	NR	NR	L3	psoas	NR	Synapse Vincent	Manual	height squared	PMI	Literature	No	No	Yes	NR
**Sui et al.**	2018	354	periampullary malignant tumors	Pancreatectomy	1 month	NR	NR	L3	total muscle area	NR	Synapse Vincent	NR	height squared	SMI	Literature	No	No	No	Yes
**Sur et al.**	2015	104	benign or malignant pancreatic disease	Pancreatectomy	NR	contrast and non-	NR	L3	psoas	NR	NR	Manual	height squared	TPAI	NR	No	No	No	NR

						contrast													
**Tankel et al.**	2018	61	pancreatic cancer	Pancreatectomy	NR	contrast	NR	L3	Psoas	NR	SW application for PACS	Manual	height squared	TPA	Literature	No	No	No	None
**Tazeoglu et al.**	2023	179	pancreatic cancer	Pancreatectomy	NR	NR	NR	L3	Psoas	NR	NR	NR	height squared	PMI	Literature	No	No	No	None
**Umetsu et al.**	2017	65	distal cholangiocarcinoma	Pancreatectomy	2 months	NR	NR	L3	Psoas	NR	NR	Manual	height squared	PMI	own cohort	No	No	No	NR
**van Vledder et al.**	2012	196	CRLM	Hepatectomy	2 months	NR	NR	L3	total muscle area	-30 to 150	MeVis Lab	Automatic	height squared	SMI	own cohort	No	No	No	NR
**Voron et al.**	2015	109	HCC	Hepatectomy	2 months	venous	NR	L3	total muscle area	-29 to 150	Advantage Window	Manual	height squared	SMI	Literature	No	No	No	NR

**Wielsoe et al.**	2024	134	benign or malignant pancreatic disease	Pancreatectomy	NR	NR	NR	L3	total muscle area	-29 to 150	SliceOMatic	NR	height squared	SMI	Literature	Yes	Yes	-	Yes
**Wu et al. (2019)**	2019	146	pancreatic cancer	Pancreatectomy	1 week	contrast	NR	L3	total muscle area	-29 to 150	ImajeJ	NR	height squared	SMI	Literature	No	No	No	NR
**Wu et al. (2023)**	2023	1172	HCC	Hepatectomy	1 month	NR	NR	L3	total muscle area	-29 to 150	ImajeJ	Manual	height squared	SMI	Literature	No	No	No	Yes
**Yasuta et al.**	2022	56	perihilar cholangiocarcinoma	Hepatectomy	1 month	NR	NR	L3	total muscle area	-29 to 150	Synapse Vincent	Manual	height squared	SMI	Literature	No	No	No	None

**Zhao et al.**	2024	302	ICC	Hepatectomy	NR	NR	NR	L3	total muscle area	-29 to 150	ImajeJ	NR	height squared	SMI	own cohort	No	No	Yes	Yes
**Zhou et al.**	2015	67	ICC	Hepatectomy	30 days	NR	NR	L3	total muscle area	-30 to 150	NR	NR	height squared	SMI	Literature	No	No	No	NR

NR, not reported; TPAI, total psoas area index; HCC, hepatocellular carcinoma; CCC, cholangiocarcinoma; GBC, gallbladder cancer; CRLM, colorectal liver metastases; HU, Hounsfield units; HUAC, Hounsfield unit average calculation; ICC, intrahepatic cholangiocarcinoma;

‡”total muscle area” was used when the muscles used to calculate the SMI were not specified in the study.
